# Atorvastatin reduces contrast media-induced pyroptosis of renal tubular epithelial cells by inhibiting the TLR4/MyD88/NF-κB signaling pathway

**DOI:** 10.1186/s12882-023-03066-9

**Published:** 2023-02-02

**Authors:** Rong-zheng Yue, Ya-juan Li, Bai-hai Su, Cong-jun Li, Rui Zeng

**Affiliations:** 1 Department of nephrology, West China Hospital, School of Clinic Medicine, Sichuan University, 610041 Chengdu, Sichuan China; 2 Department of Cardiovascular diseases, West China Hospital, School of Clinic Medicine, Sichuan University, 610041 Chengdu, Sichuan China

**Keywords:** Contrast-related acute kidney injury, Atorvastatin, Toll-like receptor 4 (TLR4), TLR4/MyD88/NF-κB

## Abstract

**Background:**

Contrast-induced acute kidney injury (CI-AKI) is the third most common cause of hospital-acquired renal failure. However, there is no effective treatment of CI-AKI, and its mechanism is unknown. Interestingly, atorvastatin has been reported to be effective in renal injury. Therefore, the aim of this study was to explore the effect and possible molecular mechanism of atorvastatin in CI-AKI.

**Methods:**

On the CI-AKI in vitro model, rat tubular epithelial cells (NRK-52E) were treated with 18 mg I/ml meglumine diatrizoate (MEG) and then pretreated with atorvastatin. pcDNA3.1-TLR4 treatment was performed to overexpress toll-like receptor 4 (TLR4) in NRK-52E cells. Cell Counting Kit-8 (CCK-8) and lactate dehydrogenase (LDH) kits were used to detect NRK-52E cell viability as well as LDH release in each group, respectively; qRT-PCR to determine mRNA expression of TLR4 in cells; western blot to detect protein expression levels of pyroptosis-related proteins (NLRP3, caspase-1, ASC, and GSDMD) and TLR4/MyD88/NF-κB signaling pathway-related proteins (TLR4, MyD88, NF-κBp65, and p-NF-κB p65) in cells.

**Results:**

MEG treatment significantly inhibited the viability of NRK-52E cells, increased pro-inflammatory factor levels and promoted pyroptosis, representing successful establishment of a rat tubular epithelial cell (NRK-52E) CI-AKI in vitro model. Notably, atorvastatin increased the activity of MEG-treated NRK-52E cells and alleviated cell injury in a concentration-dependent manner. In addition, atorvastatin significantly down-regulated the expression of TLR4 in MEG-treated NRK-52E cells. However, overexpression of TLR4 inhibited the effects of atorvastatin on increasing cell viability, alleviating cell injury, reducing pro-inflammatory factors (IL-1β, IL-6, and TNF-α) levels, and inhibiting apoptosis (by down-regulating the expression of NLRP3, caspase-1, ASC, and GSDMD). Furthermore, atorvastatin also inhibited the expression of TLR4/MyD88/NF-κB pathway-related proteins (TLR4, MyD88, and p-NF-κB p65).

**Conclusion:**

Atorvastatin can attenuate CI-AKI through increasing the activity of MEG-treated renal tubular epithelial cells, relieving cell injury, as well as inhibiting pyroptosis and inflammation. More importantly, the mechanism was achieved by inhibiting the TLR4//MyD88/NF-κB signaling pathway.

**Supplementary Information:**

The online version contains supplementary material available at 10.1186/s12882-023-03066-9.

## Introduction

Contrast-induced acute kidney injury (CI-AKI) refers to acute kidney injury caused by intravascular administration of contrast media. With an annually increasing incidence due to the wide application of contrast media in the diagnosis and treatment of clinical diseases, CI-AKI has become the third most hospital-acquired kidney injury after kidney injury caused by nephrotoxic drugs and renal perfusion insufficiency [1–3]. The main mechanism responsible for the pathophysiology of CI-AKI is a combination of the direct tubular toxicity of contrast media and inflammation. Affected by contrast media, inflammation in renal tubular epithelial cells is promoted, which induces cell apoptosis, pyroptosis and necrosis, and finally decreases renal function [4]. CI-AKI poses a significant clinical concern since it not only adversely affects the prognosis of patients but also increases their medical burden. More seriously, there are currently no specific effective preventive and therapeutic measures available. Therefore, the search for effective preventive and therapeutic measures for CA-AKI is a top priority.

Statins, selective inhibitors of HMG-CoA reductase, reduce endogenous biosynthesis of cholesterol through the inhibition of 3-hydroxy-3-methylpancreatic enzyme A (HMG-CoA) reductase [5]. Recent studies have revealed the pleiotropic effects of statins on different types of cells. In addition to significantly lowering blood lipid levels, the clinical application of stains also improves endothelial function, relieves oxidative stress and inflammation, inhibits vascular thrombosis, enhances vasomotor performance, and suppresses extracellular matrix production [6]. Based on their solubility, statins can be divided into two categories: water-soluble and fat-soluble. Atorvastatin, a class of fat-soluble statins, functions differently in various cells [7]. For example, atorvastatin inhibited NLRP3 inflammasome activation in THP-1 monocytes, as demonstrated by Yue et al. [8]; Zhou et al. discovered that atorvastatin prevented podocyte apoptosis induced by high glucose via regulating the non-coding RNA MALAT1/miR-200c/NRF2 axis [9]. On the other hand, several studies have described that atorvastatin is a key contributor to a variety of kidney injuries. In the study of Pengrattanachot et al., atorvastatin attenuated obesity-induced kidney injury through inhibiting oxidative stress and inflammation [10]. Sun et al. also elucidated that by regulating TLR4/NF-κB axis and NLRP3 inflammasome, atorvastatin could reduce renal inflammatory response induced by calcium oxalate stones [11]. Additionally, different doses of atorvastatin have been shown to inhibit contrast-induced acute tubular injury in rats by regulating the TLR4/Myd88 signaling pathway as well as inhibiting the expression of inflammatory factors [8]. Nevertheless, the effect of atorvastatin on pyroapoptosis in CI-AKI is still unclear. Therefore, a CA-AKI in vitro model of renal tubular epithelial cell was established by administration of meglumine diatrizoate (MEG) in this study. Afterwards, the effect of atorvastatin on pyroapoptosis in CI-AKI cells was explored by using a series of molecular biology and cell biology methods, in order to provide a new approach for the treatment of CI-AKI.

## Materials and methods

### Cell culture and treatment

The normal rat tubular ductal epithelial cell line NRK-52E was purchased from the National Collection of Authenticated Cell Cultures. NRK-52E cells were cultured in DMEM medium (Hyclone, USA) containing a mixture of 5% fetal bovine serum (FBS, ABW) and 100⋅ penicillin streptomycin (Solarbio, China). Then the medium was placed in a cell incubator at 37 °C with 5% CO_2_, and renewed every two days.

Atorvastatin was bought from Pfizer Inc. (USA) and prepared at 10^− 5^ mol/L using 0.9% saline. MEG as iodine-containing contrast media was purchased from Shanghai Xudong Haipu Pharmaceutical Co., Ltd. (China), with 76% iodine content of 370 mg/ml. NRK-52E cell injury model was induced by 18 mg I/ml MEG [12]. NRK-52E cells in the logarithmic growth phase were evenly seeded in 6-well plates at 3⋅10^5^ cells/well. On functional exploration, the cells were treated differently and divided into the following groups: Control group (NRK-52E cells treated only with saline), MEG group (NRK-52E cells treated with 18 mg I/ml MEG for 21 h) [12], MEG + ATV low group (NRK-52E cells treated with 18 mg I/ml MEG and with 1µM ATV for 21 h), MEG + ATV medium group (NRK-52E cells treated with 18 mg I/ml MEG and with 2µM ATV for 21 h), and MEG + ATV high group (NRK-52E cells treated with 18 mg I/ml MEG and with 4µM ATV for 21 h).

Negative pcDNA3.1 (NC) and overexpressed pcDNA3.1-toll-like receptor 4 (TLR4) were designed and synthesized by Guangzhou RiboBio Co., Ltd. (RiboBio). In the validation experiment, NRK-52E cells were treated and grouped as follows: Control group (NRK-52E cells treated only with saline), MEG group (NRK-52E cells treated with 18 mg I/ml MEG for 21 h), MEG + ATV + NC group (NRK-52E cells treated with 18 mg I/ml MEG, with 4µM ATV for 21 h and transfected with negative pcDNA3.1), and MEG + ATV + TLR4 group (NRK-52E cells treated with 18 mg I/ml MEG, with 4µM ATV for 21 h and transfected with pcDNA3.1-TLR4).

### Cell counting Kit-8 (CCK-8)

NRK-52E cells in the logarithmic growth phase were inoculated into 96-well plates at 3⋅10^3^ cells/100µl/well. After cell adherence, NRK-52E cells received drug stimulation according to the experimental design described in 1.1. The cell viability after 0 and 24 h of drug treatment was measured following the instructions of CCK-8 kit (Solarbio, China), respectively. Specifically, 10 µl of CCK-8 solution was added to the wells and incubated in an incubator at 37 °C for 2 h. Finally, the absorbance value at 450 nm was measured with a microplate reader, and cell viability was calculated based on the OD value. Cell viability (%) = [A (added) -A (blank)]/[A (0 added) -A (blank)] × 100, A (added): absorbance of wells with cells, CCK-8 solution, and drug solution; A (blank): absorbance of wells with medium and CCK-8 solution without cells; A (0 added): absorbance of wells with cells and CCK-8 solution without drug solution.

### Lactate dehydrogenase (LDH) release assay

Appropriate amounts of NRK-52E cells were inoculated into 96-well cell culture plates, and different drug stimulation was performed after cell adherence. Upon the cell confluence of 80–90%, LDH release was detected using a LDH cytotoxicity assay kit (Beyotime, China). Subsequently, 120 µl of cell culture supernatant was added to a new 96-well plate and mixed with 60 µl of LDH detection solution, followed by 30-min incubation in a cell incubator. Optical density (OD) at 490 nm was measured with a microplate reader, and ultimately the percentage of LDH release was calculated. Percentage of LDH release = (OD experimental group − OD Control group)/(ODmax − OD Control group) × 100%.

### Enzyme-linked immunosorbent assay (ELISA)

The culture medium supernatant of NRK-52E cells was collected and centrifuged at 1000 × g for 10 min at 4 °C. Subsequent to the removal of cell debris and other impurities, the supernatant was aliquoted into small EP tubes and stored at − 20 °C to avoid repeated freezing and thawing. The levels of IL-1β, IL-6 and TNF-α in the cell supernatant were then measured under the instructions of the corresponding ELISA assay kit (Cusabio Biotech Co., Ltd, China).

### Quantitative real-time polymerase chain reaction (qRT-PCR)

Using the Trizol method (Sigma, USA), total RNA was extracted from NRK-52E cells, and RNA concentration was determined by Nanodrop. The extracted RNA was reverse transcribed into cDNA using the PrimeScript RT kit (Takara, Japan). Later, the obtained cDNA was employed as a template for qRT-PCR analysis with the help of the SYBR Green Premix kit (Vazyme, China). Taking β-actin as an internal control gene, the relative expression level of TLR4 was calculated. The primer sequences used are shown in Table 1.

**Table 1:** Primer sequence used in qRT-PCR

Gene name	Forward Primer (5’ to 3’)	Reverse Primer (5’ to 3’)
TLR4	CGTATACGACGAGTTCCAGT	GGACTTGTTGGACGTCGAGA
β-actin	GGAGATTACTGCCCTGGCTCCTA	GACTCATCGTACTCCTGCTTGCTG

### Western blot

NRK-52E cells were lysed by RIPA protein lysate containing PMSF (Beyotime, China). Following the disruption by sonication in an ice bath and centrifugation at 10,000 rpm and 4 °C for 15 min, total protein supernatants were collected. Through the use of a BCA protein assay kit (Beyotime, China), the concentration of total protein was determined. After that, the protein was denatured at 95 °C for 15 min with 5⋅ loading buffer. SDS-PAGE protein electrophoresis (80 V for 30 min followed by 120 V for 1 h) was performed to separate out 20 µg of total protein. Subsequently, the separated protein was transferred from the gel to a polyvinylidene fluoride (PVDF, Millipore, USA) membrane which was later blocked with 3% BSA solution for 30 min. Next, the membrane was incubated overnight at 4 °C in a shaker with the primary antibodies (Anti-TLR4, ab22048, abcam; Anti-NLRP3, NBP2-12446, Novus Biologicals; Anti-caspase-1, NB100-56565, Novus Biologicals; Anti-ASC, ab180799, Abcam; Anti-GSDMD, 39,754, Cell Signaling Technology; Anti-MyD88, 4283, Cell Signaling Technology; Anti-p-NF-κB p65 (Ser536), 3033, Cell Signaling Technology; Anti-NF-κB p65, ab16502, Abcam; Anti-β-actin, 4970, Abcam). After three rinses of the membrane with TBST, the second antibodies (ZB2301, ZB2305, ZSGB-BIO, China) were supplemented and incubated at ambient temperature for 1 h. Again, the membrane was washed with TBST three times, and then ECL hypersensitive luminescence solution (Beyotime, China) was applied for exposure in a chemiluminescence imager. Besides, the gray values of the bands were quantitatively analyzed using Image Pro Plus software (with β-actin as an internal control protein).

### Statistical analysis

All data were expressed as mean ± standard deviation (SD), and SPSS 26 software was used for statistical analysis. One-way analysis of variance (ANOVA) was used to compare the data between multiple groups. *P* < 0.05 was deemed as the judgment criteria for statistically significant differences.

## Results

### Atorvastatin increases viability and reduces injury of NRK-52E cells treated with meglumine diatrizoate

**Fig. 1:**
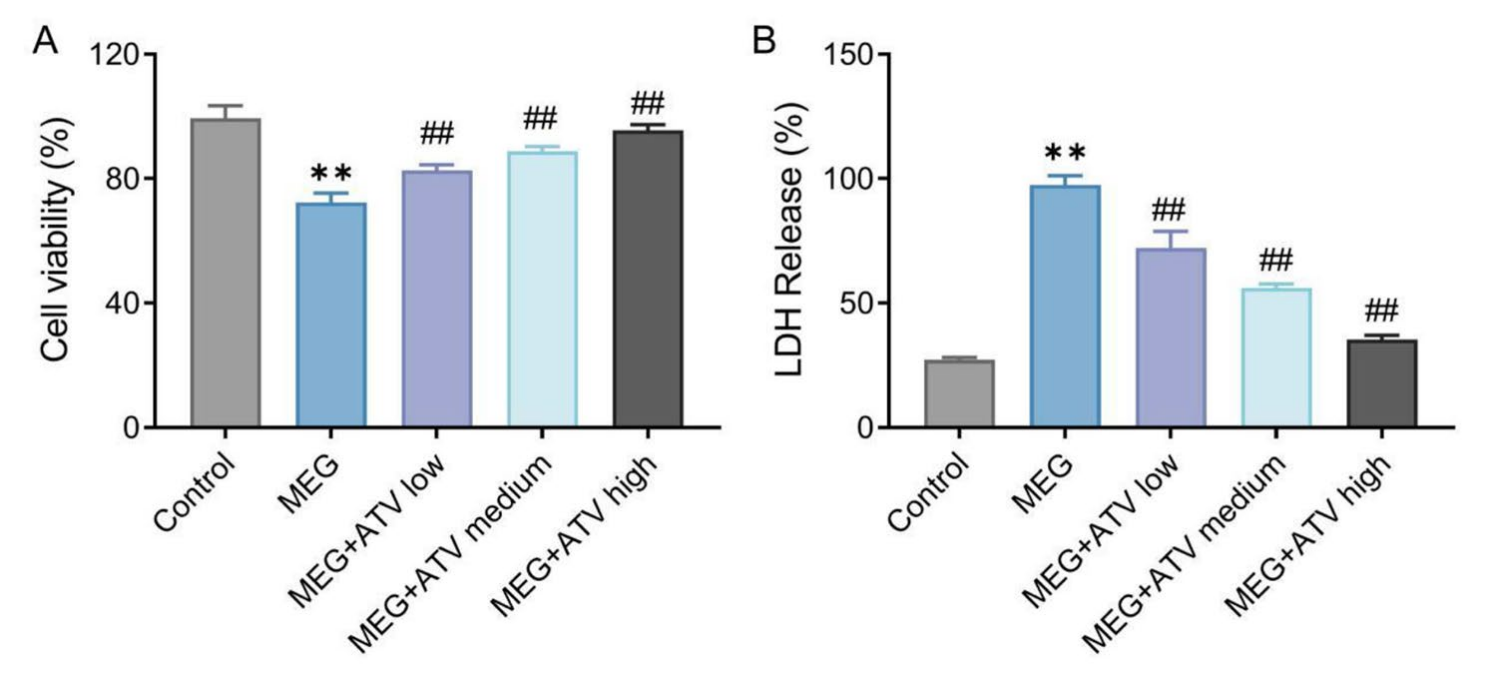
Atorvastatin increases viability and decreases cell injury in NRK-52E cells treated with MEG. A, CCK-8 results of NRK-52E cell viability in each group. B, The activity of LDH in NRK-52E cells of each group detected by LDH cytotoxicity assay kit. **P < 0.01 vs. Control. ##*P* < 0.01 vs. MEG. MEG, meglumine diatrizoate; LDH, lactate dehydrogenase

In order to observe the effect of atorvastatin on MEG-induced decrease in NRK-52E cell viability and increase in LDH activity, we used 18 mg I/ml MEG to induce NRK-52E cell injury and tested NRK-52E cell viability and LDH activity by CCK-8 and LDH cytotoxicity assay kits. The test results showed that, compared with the control group, the viability of cells in the MEG group was significantly reduced (*P* < 0.01); different concentrations of atorvastatin could obviously increase the viability of NRK-52E cells in a concentration-dependent manner (*P* < 0.01, Fig. 1 A). In addition, MEG was able to improve LDH activity in NRK-52E cells, while ATV treatment notably inhibited LDH activity in NRK-52E cells (*P* < 0.01) in a concentration-dependent manner (Fig. 1B). The above results proved that the CI-AKI model was successfully constructed, and atorvastatin increased the viability and decreased cell injury of MEG-treated NRK-52E cells.

### Atorvastatin down-regulates the expression of toll-like receptor 4 in meglumine diatrizoate-treated NRK-52E cells

**Fig. 2:**
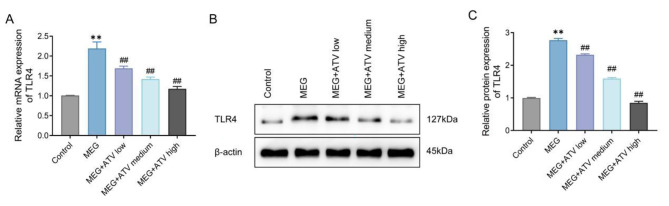
Atorvastatin down-regulates TLR4 expression in MEG-treated NRK-52E cells. A, mRNA expression levels of TLR4 in NRK-52E cells from each group detected by qRT-PCR. B/C, Protein expression levels of TLR4 in NRK-52E cells from each group detected by western blot (Cut Western Blot Images). **P < 0.01 vs. Control. ##*P* < 0.01 vs. MEG. TLR4, toll-like receptor 4; MEG, meglumine diatrizoate

The results of qRT-PCR and western blot showed that mRNA and protein expression levels of TLR4 were obviously up-regulated in NRK-52E cells in the MEG group compared with the control group, while those levels were significantly down-regulated in MEG + ATV cells compared with the MEG group in a concentration-dependent manner (*P* < 0.01, Fig. 2 A-C). It was logical to speculate that atorvastatin treatment significantly down-regulated TLR4 expression in MEG-treated NRK-52E cells.

### Atorvastatin increases the viability and reduces cell injury of meglumine diatrizoate-treated NRK-52E cells by downregulating the expression of toll-like receptor 4

**Fig. 3:**
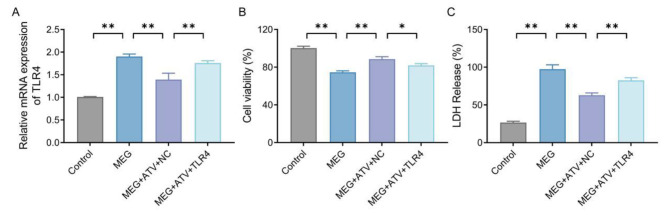
Atorvastatin increases the viability and relieves cell injury of MEG-treated NRK-52E cells by down-regulating TLR4. A, mRNA expression levels of TLR4 in NRK-52E cells from each group detected by qRT-PCR. B, the viability of NRK-52E cells in each group detected by CCK-8. C, LDH activity of NRK-52E cells in each group detected by kit. *P < 0.05 and **P < 0.01. MEG, meglumine diatrizoate; TLR4, toll-like receptor 4; LDH, lactate dehydrogenase

In order to investigate whether atorvastatin can improve the viability and reduce cell injury of MEG-treated NRK-52E cells by down-regulating TLR4 expression, we transfected TLR4 overexpression plasmid (pcDNA3.1-TLR4) to elevate the level of TLR4 in NRK-52E cells. Later, qRT-PCR results indicated that the mRNA expression level of TLR4 was notably increased in MEG cells compared with the control group; the expression level of TLR4 was much lower in MEG + ATV cells than that in the MEG group; and the mRNA expression level of TLR4 was markedly elevated in MEG + ATV + TLR4 NRK-52E cells relative to MEG + ATV + NC group (*P* < 0.01, Fig. 3 A). As for the findings of cell viability and LDH activity assay, in comparison to the MEG group, the MEG + ATV + NC group displayed an obvious increased viability of cells and decreased LDH activity; a significant decrease in the viability of NRK-52E cells (*P* < 0.05) and a considerable increase in LDH activity (*P* < 0.01) were discovered in MEG + ATV + TLR4 group compared with the MEG + ATV + NC, (Fig. 3B/C). Overall, overexpression of TLR4 inhibited the effects of atorvastatin on the viability and cell injury of MEG-treated NRK-52E cells.

### Atorvastatin reduces proinflammatory factor levels in meglumine diatrizoate-treated NRK-52E cells by down-regulating toll-like receptor 4

**Fig. 4:**
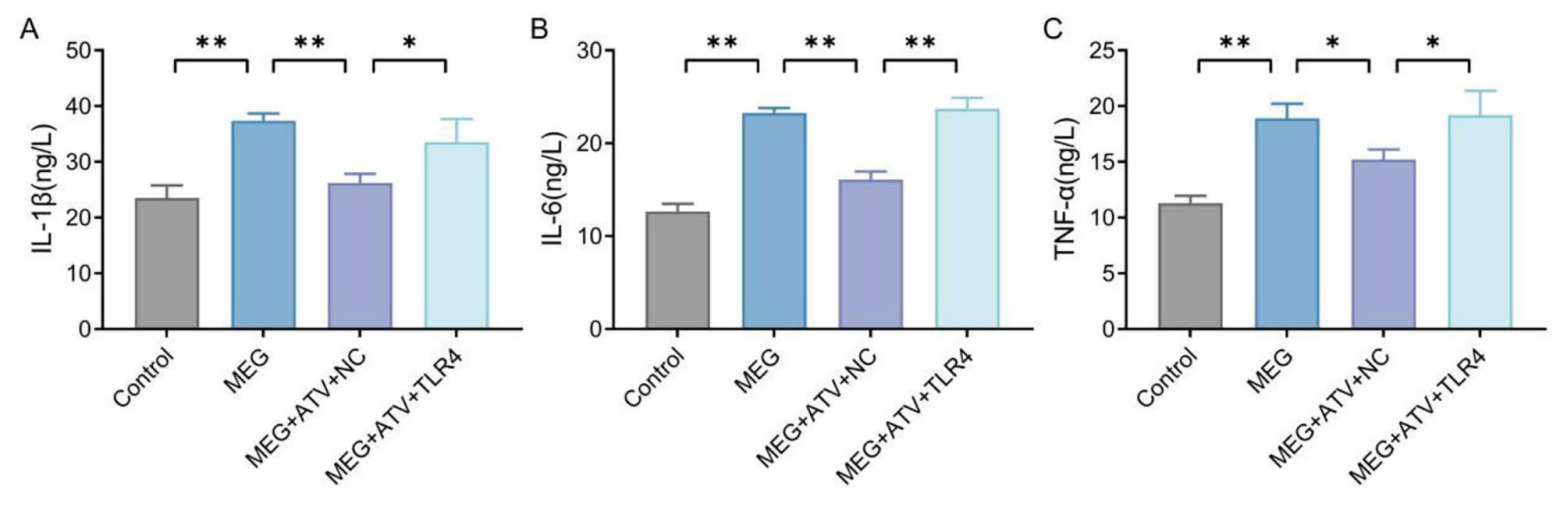
Atorvastatin decreases proinflammatory factor levels in MEG-treated NRK-52E cells by downregulating TLR4. A-C, ELISA results of the expression levels of pro-inflammatory cytokines IL-1β (A), IL-6 (B) and TNF-α (C) in NRK-52E cells of each group. *P < 0.05 and **P < 0.01. MEG, meglumine diatrizoate; TLR4, toll-like receptor 4; ELISA, enzyme-linked immunosorbent assay

With the purpose of determining whether atorvastatin was able to alleviate the inflammatory response in MEG-treated NRK-52E cells, we examined the expression of pro-inflammatory cytokines IL-1β, IL-6, and TNF-α in NRK-52E cells using ELISA. The ELISA results revealed that compared with the Control group, the expression levels of IL-1β, IL-6 and TNF-α in the cells of the MEG group were considerably elevated; while compared with the MEG group, the expression levels of IL-1β, IL-6 and TNF-α in the cells of the MEG + ATV + NC group were remarkedly reduced; relative to the MEG + ATV + NC group, those levels of the MEG + ATV + TLR4 group were notably up-regulated (*P* < 0.05, Fig. 4 A-C). In a nutshell, overexpression of TLR4 could reverse the inhibitory effect of MEG on inflammation in NRK-52E cells.

### Atorvastatin inhibits pyroptosis in meglumine diatrizoate-treated NRK-52E cells by down-regulating the expression of toll-like receptor 4

**Fig. 5:**
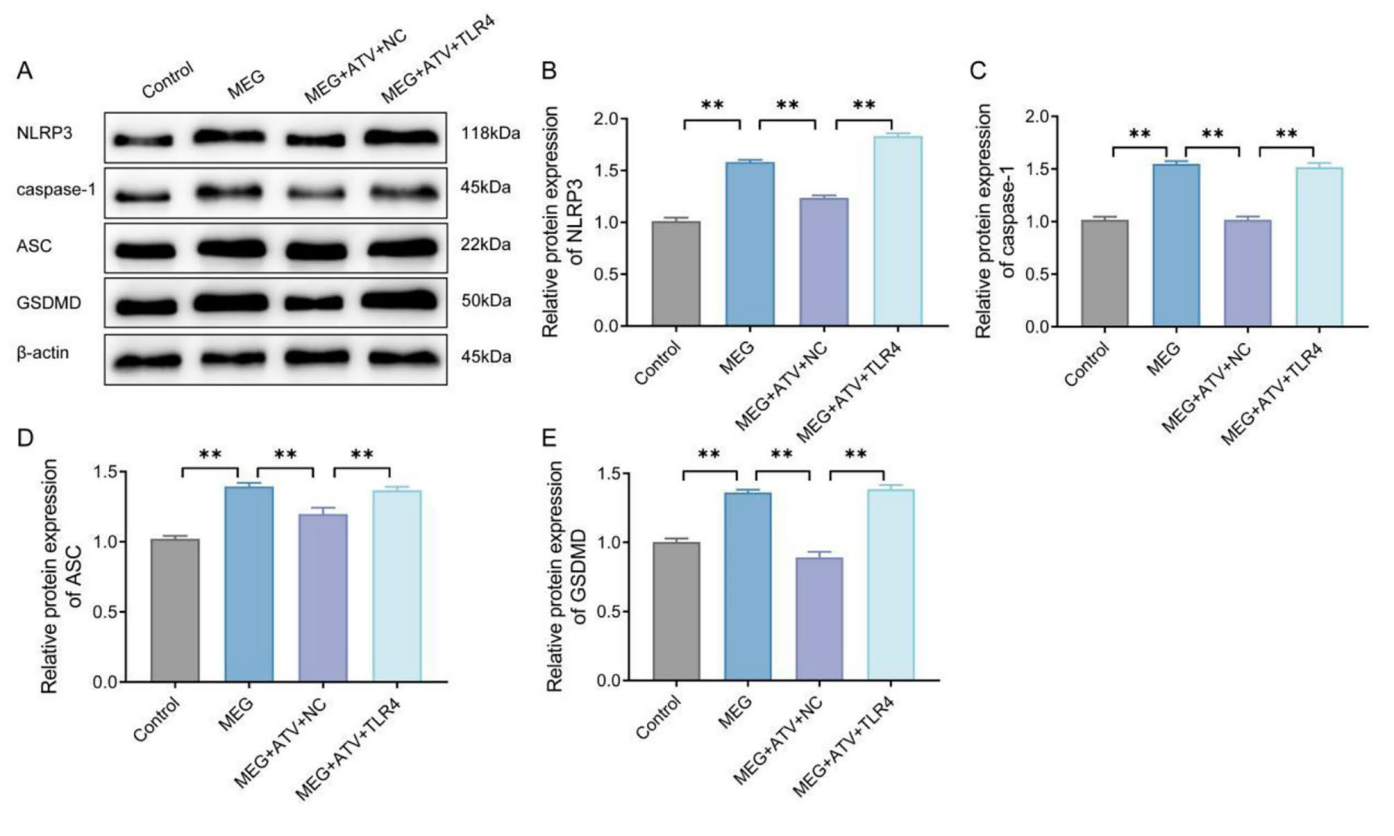
Atorvastatin inhibits pyroptosis in MEG-treated NRK-52E cells by downregulating TLR4 expression. A, Western blot results of the expression levels of pyroptosis-related proteins (NLRP3, caspase-1, ASC and GSDMD) in NRK-52E cells in each group (Cut Western Blot Images). B-E, Image pro plus quantification of the gray value of the target protein and calculation of the relative expression of the target protein using β-actin as an internal control. **P < 0.01. MEG, meglumine diatrizoate; TLR4, toll-like receptor 4

Previous studies have indicated that contrast media are capable of inducing pyroptosis in renal tubular epithelial cells [13, 14]. In this study, we examined the expression levels of pyroptosis-related proteins (NLRP3, caspase-1, ASC and GSDMD) using western blot to investigate whether atorvastatin has an effect on tubular pyroptosis. In contrast to the control group, the protein expression levels of NLRP3, caspase-1, ASC and GSDMD in NRK-52E cells treated with MEG were significantly lifted (*P* < 0.01); atorvastatin effectively reduced the protein levels of NLRP3, caspase-1, ASC and GSDMD in MEG-treated NRK-52E cells (*P* < 0.01); compared with the MEG + ATV group, the protein levels of NLRP3, caspase-1, ASC and GSDMD were noticeably risen in MEG + ATV + TLR4 group (*P* < 0.01) (Fig. 5 A-E). The above results suggested that overexpression of TLR4 could suppress the inhibitory effect of atorvastatin on pyroptosis in MEG-treated NRK-52E cells.

### Atorvastatin inhibits TLR4/MyD88/NF-κB signaling pathway activity in meglumine diatrizoate-treated NRK-52E cells

**Fig. 6:**
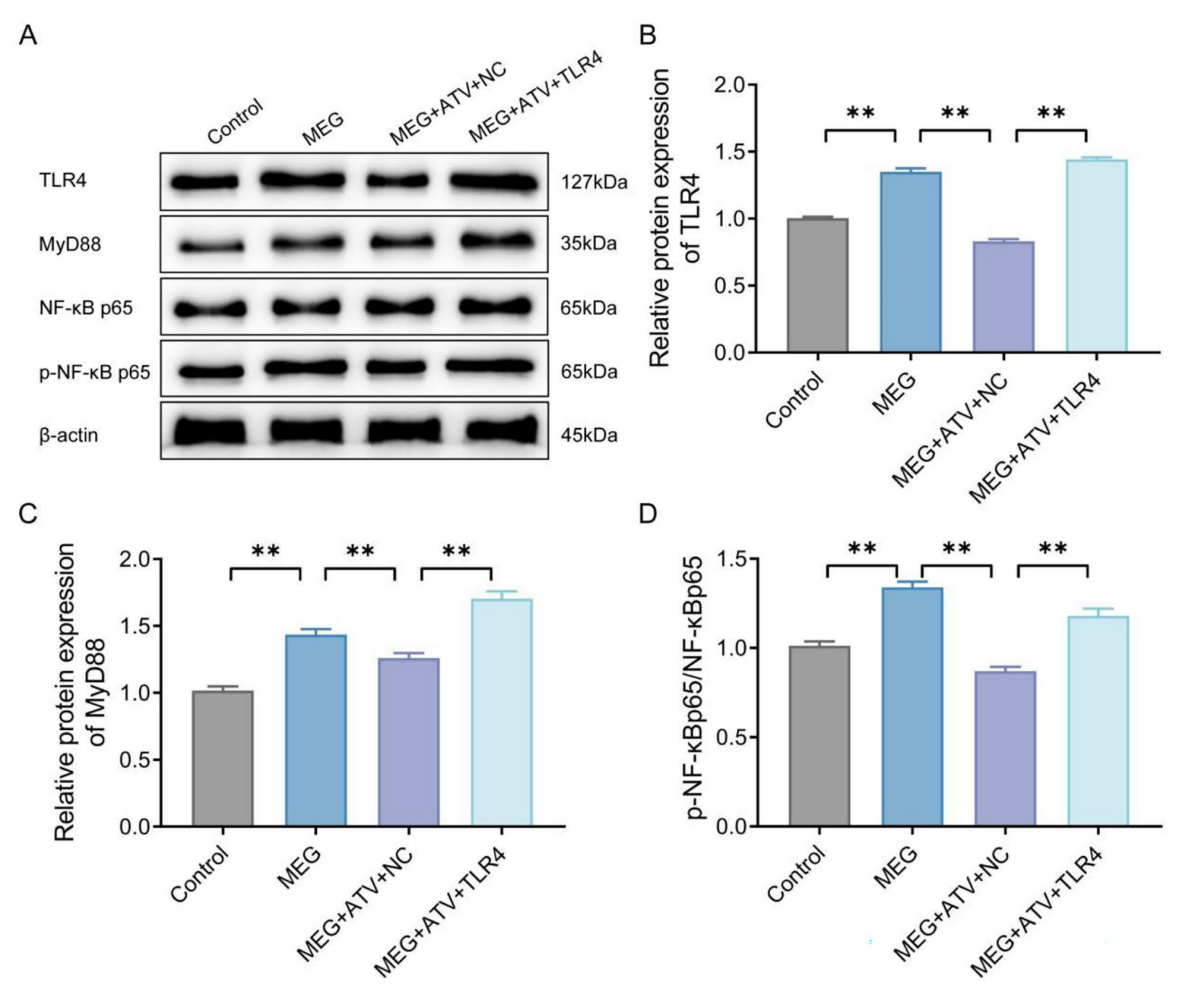
Atorvastatin inhibits TLR4/MyD88/NF-κB signaling pathway activity in MEG-treated NRK-52E cells. A, Western blot results of the expression levels of TLR4/MyD88/NF-κB signaling pathway-related proteins (NLRP3, caspase-1, ASC and GSDMD) in NRK-52E cells of each group (Cut Western Blot Images). B-D, Image pro plus quantification of the gray value of the target protein and calculation of the relative expression of the target protein using β-actin as an internal control. **P < 0.01. MEG, meglumine diatrizoate; TLR4, toll-like receptor 4

As described in previous studies, TLR4/MyD88/NF-κB contributes to inflammation, apoptosis, and pyroptosis in various organ tissue injuries such as lung injury and myocardial injury [15–17]. Moreover, atorvastatin was discovered to increase cell viability and inhibite inflammation and pyroptosis by inhibiting TLR4 in MEG-treated NRK-52E cells. Accordingly, we speculated that atorvastatin may exert the above effects through the TLR4/MyD88/NF-κB pathway. Subsequently, we detected the expression of TLR4/MyD88/NF-κB signaling pathway-related proteins in NRK-52E cells of each group by western blot. The findings included that the protein levels of TLR4, MyD88, p-NF-κB p65 and the ratio of p-NF-κB p65/NF-κB p65 were much higher in the MEG group than those in the control group; while the protein levels of TLR4, MyD88, p-NF-κB p65 and the ratio of p-NF-κB p65/NF-κB p65 were much lower in the MEG + ATV + NC group than those in the MEG group; in addition, the protein levels of TLR4, MyD88, p-NF-κB p65 and the ratio of p-NF-κB p65/NF-κB p65 were quite higher in the MEG + ATV + TLR4 group than those in the MEG + ATV + NC group (*P* < 0.01, Fig. 6 A-D). Briefly speaking, atorvastatin functioned in MEG-treated NRK-52E cells through the TLR4/MyD88/NF-κB signaling pathway.

## Discussion

With the development of interventional techniques, recent years have witnessed the increasing incidence of CA-AKI. CA-AKI has been reported to account for 11% of all hospital-acquired kidney injuries. Unfortunately, no adjuvant drugs have been discovered to be effective in preventing or treating CI-AKI. However, some articles have indicated that αKlotho protein can prevent CA-AKI development by inhibiting NLRP3 inflammasome-induced pyroptosis [18, 19]. Some scholars have also proposed that Nrf2 agonists, such as sulforaphane, can be adopted to treat contrast-induced kidney injury. Especially, sulforaphane has a more significant therapeutic effect in the treatment of diabetes [12]. Although the mechanism of CI-AKI is not fully understood, a series of studies have revealed that contrast media exert toxic effects on renal tubules [20]. In our study, the CI-AKI in vitro model of NRK-52E cells was induced by MEG treatment. The discovery was that MEG treatment resulted in significantly decreased cell viability of NRK-52E, increased LDH release as well as levels of inflammatory factors and pyroptosis. Likewise, Khaleel et al. also established a NRK-52E cell injury model with significantly reduced cell viability by MEG treatment [12].

N-acetylcysteine, nicorandil, and statins have been proven to protect kidneys in patients with kidney injuries, but their mechanisms remain to be further clarified [21–23]. Accordingly to the results of a small randomized trial and meta-analysis, specific drugs, such as N-acetylcysteine, ascorbic acid, aminophylline, trimetazidine, fenoldopam, etc., were effective in the treatment of CI-AKI, but they failed to prevent or treat CI-AKI in a large randomized trial. However, statins may exert renoprotective effects through multiple mechanisms, including inhibition of contrast media absorbed into tubular cells, reduction of endothelial dysfunction and oxidative stress, anti-inflammation, anti-proliferation of mesangial cells, and protection of podocyte [24]. Lin et al. claimed that atorvastatin significantly improved renal dysfunction and morphological changes as well as inflammation, apoptosis, and excessive oxidative stress in CI-AKI rats through in vivo experiments. Similarly, the cell experiment they performed also displayed the inhibitory effect of atorvastatin on contrast-induced apoptosis and inflammation by endogenous up-regulation [25]. The results of an in vivo experiment conducted by Yue et al. demonstrated that different doses of atorvastatin could alleviate kidney injury as well as reduce apoptosis and inflammatory factor levels (IL-1β, IL-6 and MCP-1) in the kidneys of CI-AKI rats [8]. In line with their findings, we also found that atorvastatin significantly increased cell viability, attenuated cell injury, lowered inflammatory factor levels, and inhibited pyroptosis through in vitro cell experiments. So far, no study has shown that atorvastatin can inhibit pyroptosis in CI-AKI. Nonetheless, a study by Left et al. revealed that atorvastatin played a protective role in high glucose-induced podocyte pyroptosis by regulating MALAT1/miR-200c/NRF2 activity [9]. In addition, atorvastatin reduced early brain injury by inhibiting pyroptosis and cellular inflammation, as described by Chen et al. [26]. Collectively, atorvastatin alleviates CI-AKI by increasing tubular cell activity as well as inhibiting pyroptosis and inflammation.

**Fig. 7:**
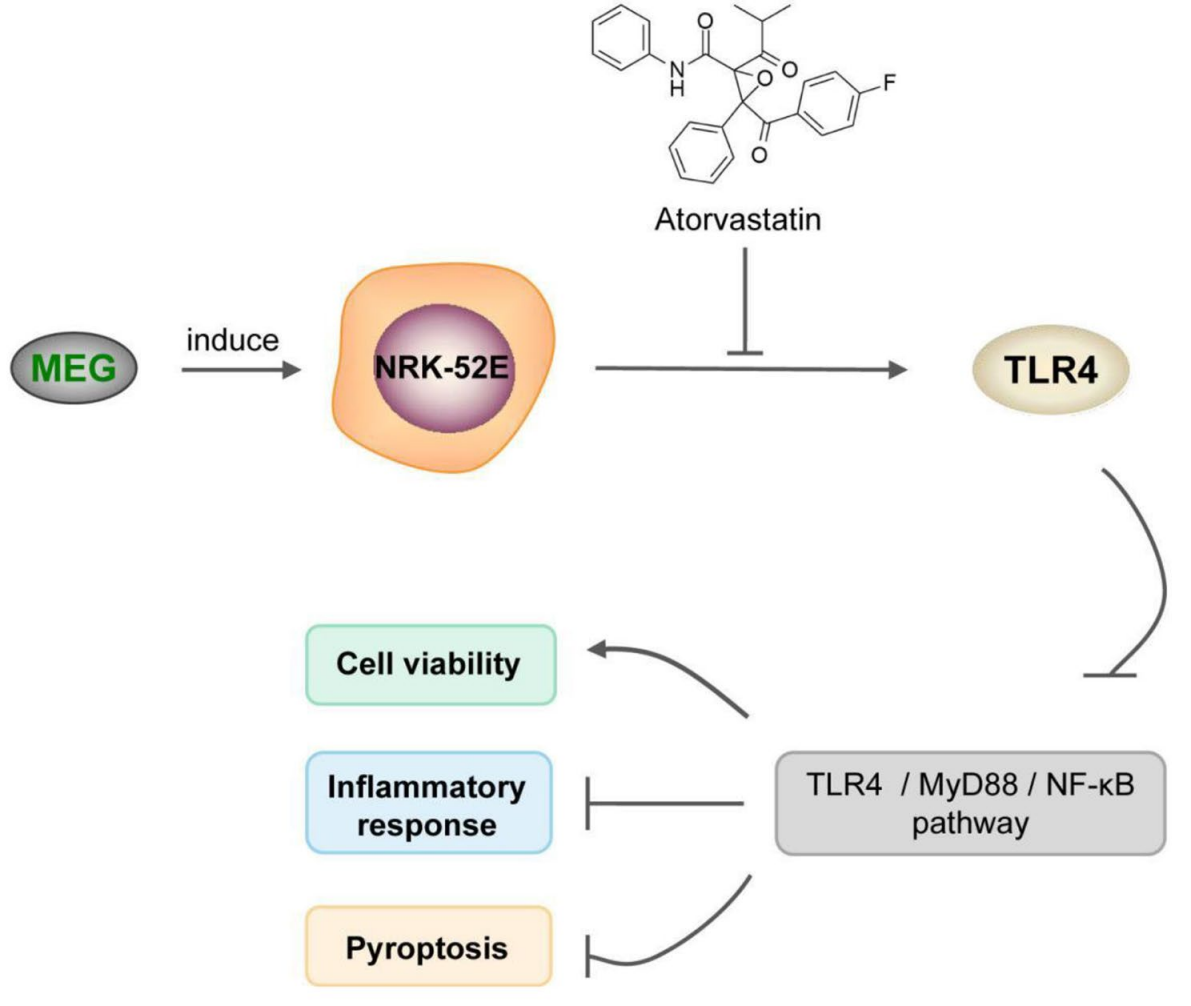
Schematic diagram of atorvastatin on contrast medium induced pyroptosis of renal tubular epithelial cells. MEG activates TLR4/MyD88/NF-κB signaling pathway in NRK-52E cells, while atorvastatin inhibits TLR4/MyD88/NF-κB signal pathway activity, thereby reducing MEG induced NRK-52E cell damage, inflammatory response and cell scorch. MEG, meglumine diatrizoate; TLR4, toll-like receptor 4

In the mechanistic exploration of this study, atorvastatin obviously down-regulated TLR4 expression in MEG-treated NRK-52E cells, and overexpression of TLR4 greatly inhibited the protective effect of atorvastatin in the CI-AKI model. This suggests that atorvastatin can attenuate MEG-treated NRK-52E cell injury by downregulating TLR4 expression. Furthermore, the pathway exploration presented that MEG activated TLR4/MyD88/NF-κB signaling pathway in NRK-52E cells, while atorvastatin inhibited TLR4/MyD88/NF-κB signaling pathway activity. Hence, it is logical to speculate that atorvastatin may improve MEG-induced pyroptosis in tubular epithelial cells by inhibiting TLR4/MyD88/NF-κB signaling pathway and thus alleviate CI-AKI (Fig. 7). Contrast media have been demonstrated in several studies to cause inflammation and apoptosis of HK-2 in human renal cortical proximal tubular epithelial cells by activating the TLR4/MyD88/NF-κB axis [27–30]. However, our study did not validate the mechanism of this pathway. Therefore, further experiments are required to verify that atorvastatin can improve cell activity as well as inhibit pyroptosis and inflammation in MEG-treated NRK-52E cells through this pathway. Additionally, necessary in vivo experiments were lacking in our study.

## Conclusion

Taken together, atorvastatin can reduce MEG-induced NRK-52E cell activity, inflammation, and pyroptosis through inhibiting TLR4, which may be achieved by inhibiting TLR4/MyD88/NF-κB signaling pathway. Of note, this study provides a theoretical basis for the application of statins in the clinical treatment of CI-AKI.

## Electronic supplementary material

Below is the link to the electronic supplementary material.

Supplementary Material 1

## Data Availability

Datasets used in this article are available from corresponding author on reasonable request.
