# Supplementary figures and images for: Atorvastatin reduces contrast media-induced pyroptosis of renal tubular epithelial cells by inhibiting the TLR4/MyD88/NF-κB signaling pathway
Source: BMC Nephrol. 2023 Feb 2;24:25. doi: 10.1186/s12882-023-03066-9 (PMC9893683; doi:10.1186/s12882-023-03066-9)

Figure 2B


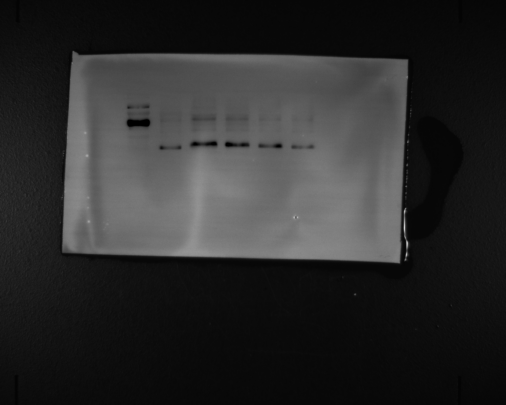


TLR4


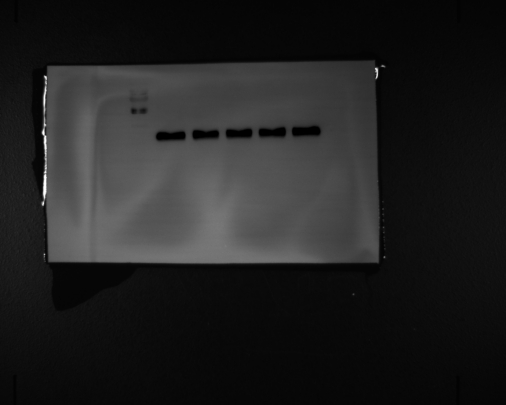


β-actin

Figure 5A


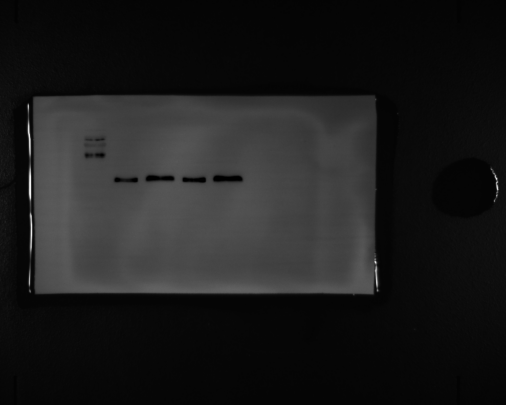


NLRP3


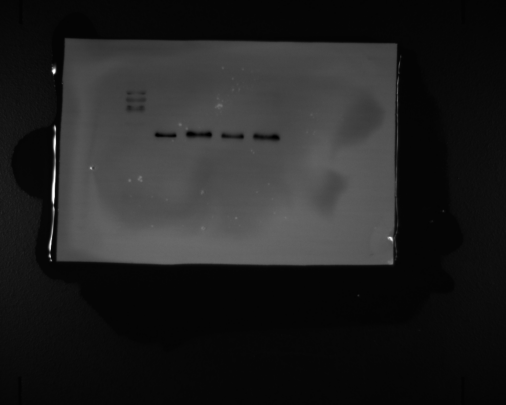


caspase-1


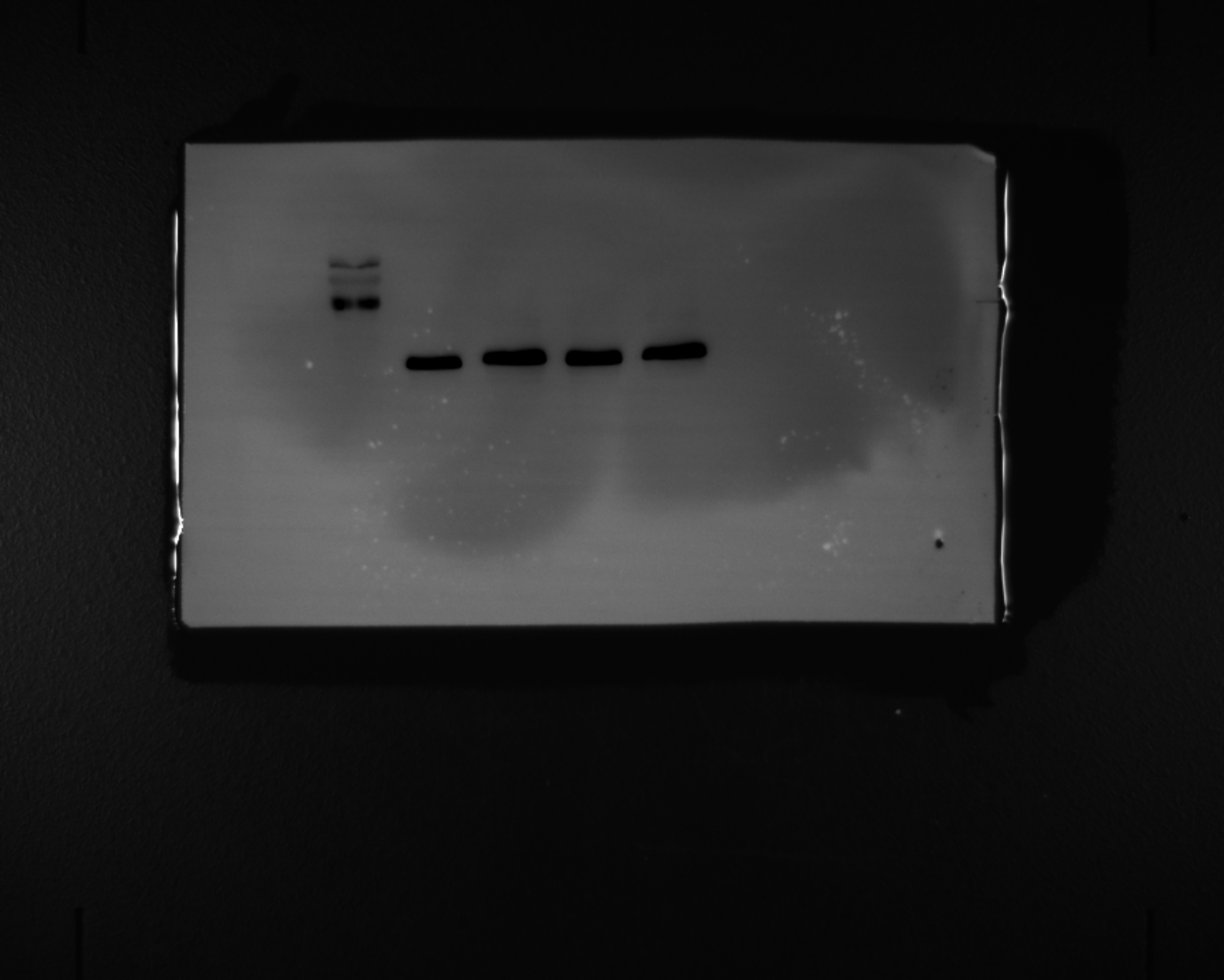


ASC


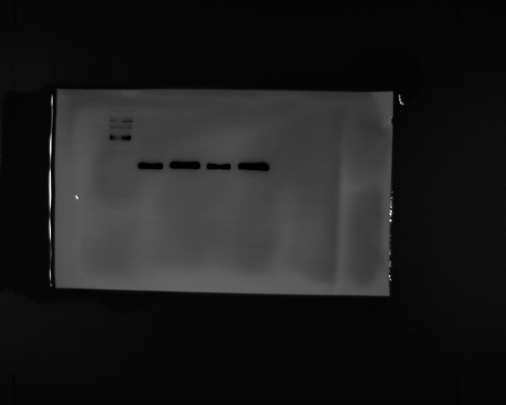


GSDMD


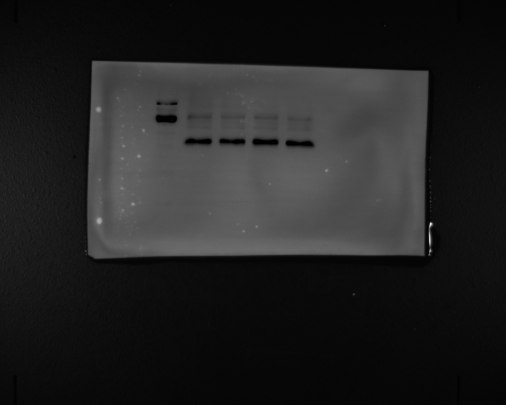


β-actin

Figure 6A


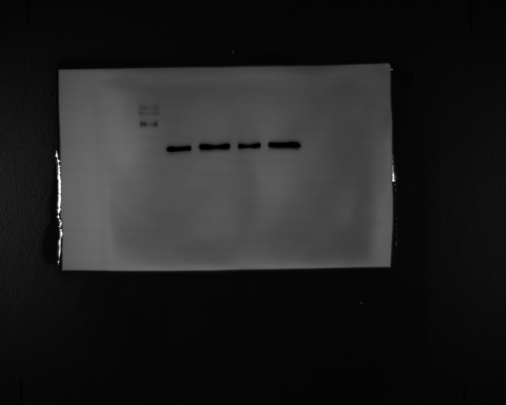


TLR4


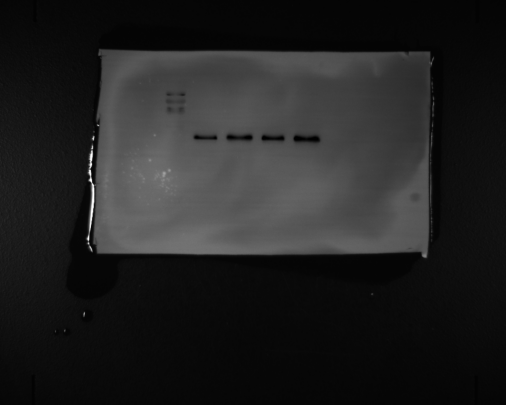


MyD88


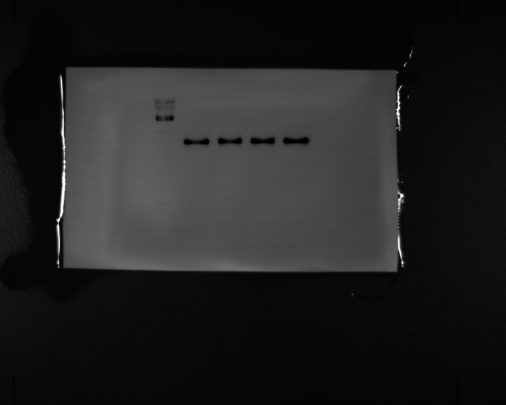


NF-κBp65


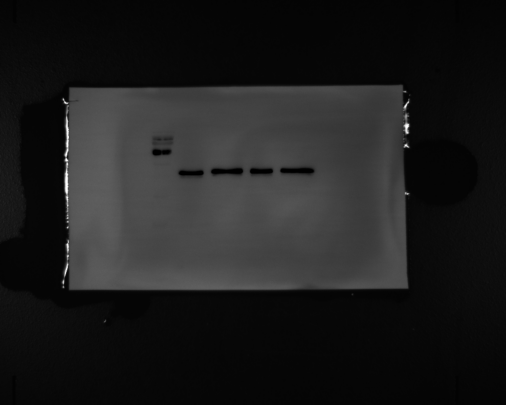


p-NF-κBp65


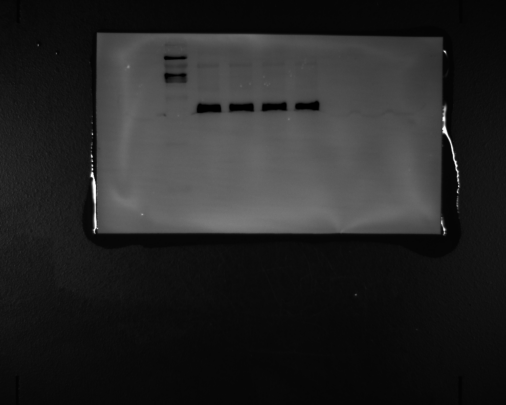


β-actin

Supplement: Supplementary file 1 — Supplementary Material 1 [file 12882_2023_3066_MOESM1_ESM.docx]
